# Discovery of Dimeric Arylsulfonamides as Potent ADAM8 Inhibitors

**DOI:** 10.1021/acsmedchemlett.1c00411

**Published:** 2021-10-08

**Authors:** Doretta Cuffaro, Caterina Camodeca, Tiziano Tuccinardi, Lidia Ciccone, Jörg W. Bartsch, Tanja Kellermann, Lena Cook, Elisa Nuti, Armando Rossello

**Affiliations:** † Department of Pharmacy, University of Pisa, via Bonanno 6, 56126 Pisa, Italy; ‡ Department of Neurosurgery, Marburg University, Baldingerstrasse, 35033 Marburg, Germany

**Keywords:** ADAM8 dimerization, bifunctional inhibitors, arylsulfonamide hydroxamates, anticancer drugs

## Abstract

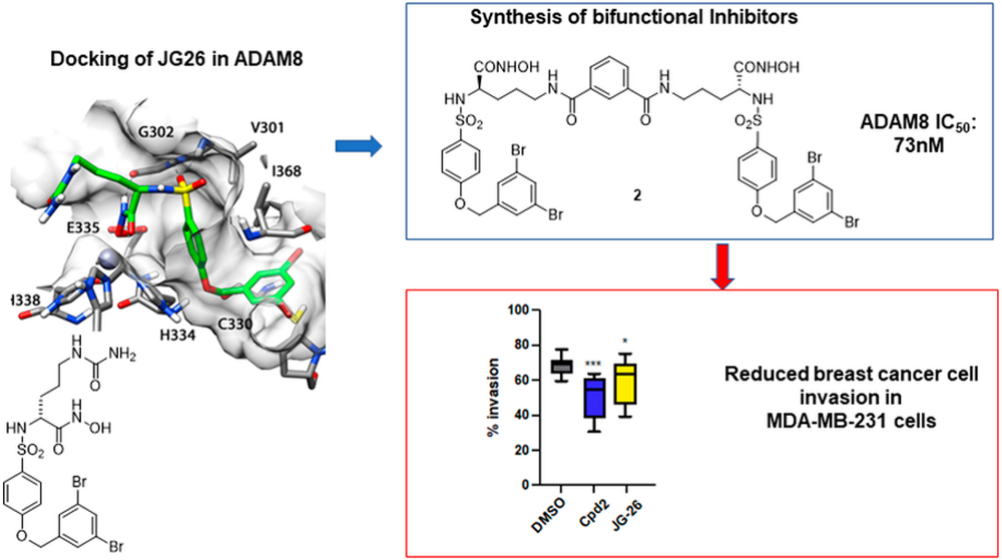

The metalloproteinase ADAM8 is upregulated in several cancers but has a dispensable function under physiological conditions. In tumor cells, ADAM8 is involved in invasion, migration, and angiogenesis. The use of bivalent inhibitors could impair migration and invasion through the double binding to a homodimeric form of ADAM8 located on the cell surface of tumor cells. Herein we report the rational design and synthesis of the first dimeric ADAM8 inhibitors selective over ADAM10 and matrix metalloproteinases. Bivalent derivatives have been obtained by dimerizing the structure of a previously described ADAM17 inhibitor, JG26. In particular, derivative **2** was shown to inhibit ADAM8 proteolytic activity *in vitro* and in cell-based assays at nanomolar concentration. Moreover, it was more effective than the parent monomeric compound in blocking invasiveness in the breast cancer MDA-MB-231 cell line, thus supporting our hypothesis about the importance of inhibiting the active homodimer of ADAM8.

A Disintegrin and Metalloproteinase 8 (ADAM8) is a transmembrane protein belonging to the metzincin superfamily of metalloproteases. As a multidomain enzyme, ADAM8 is constituted by an N-terminal prodomain followed by a metalloproteinase (MP), a disintegrin (DIS), a cysteine-rich, epidermal-growth-factor (EGF)-like transmembrane domain, and a cytoplasmic tail.^1^ ADAM8 is activated by autocatalysis in the trans-Golgi network and, for *in vivo* activity, requires the homophilic multimerization of at least two ADAM8 monomers on the cell membrane. In particular, the dimerization depends on DIS domain interactions.^2^ Different from other family members, such as ADAM17 and ADAM10,^3^ ADAM8 is not essential under physiological conditions, but it is upregulated in inflammatory processes and in several cancers.^4^ Through its proteolytic activity, it is responsible for the cleavage of cell surface proteins (ectodomain shedding) and the degradation of extracellular matrix (ECM) components. The sheddase function depends on the MP domain, where a zinc ion is present in the active site and is essential for catalytic activity. Among its substrates are cytokine receptors, cell adhesion molecules, and immune modulators, such as the low affinity IgE receptor CD23. Once upregulated in cancer, ADAM8 proteolytic activity can promote tumorigenesis by inducing angiogenesis and metastasis.^5^ There is also evidence of nonproteolytic functions of ADAM8, mainly due to the interaction of its DIS domain with β1-integrin on the cell surface. This interaction is responsible for the intracellular activation of the MAPK signaling pathway involved in the chemoresistance of tumoral cells.

Owing to its dispensable function under physiological conditions, ADAM8 has been considered a promising drug target for cancer therapy, whose inhibition could give fewer side effects than ADAM10/17 inhibitors.^6^ In particular, ADAM8 has been recently validated as a drug target in pancreatic,^7^ liver,^8^ and breast cancer.^9^ Unfortunately, the development of potent and selective ADAM8 inhibitors has been hampered by the high homology among matrix metalloproteinase (MMP) and ADAM catalytic sites. Broad-spectrum metalloprotease inhibitors such as marimastat have failed in clinical trials for cancer due to muscoloskeletal side effects and toxicity.^10^ On the contrary, a selective inhibition of ADAM8 has been achieved by Schlomann et al.^7^ using an exosite inhibitor, a short cyclic peptide designed to block ADAM8 activation by interacting with the DIS domain. A library of peptidomimetic analogues of cyclo(RLsKDK) has been developed by Yim et al.^11^ but a more stable nonpeptide compound, able to inhibit ADAM8 proteolytic activity, is still lacking.

Dimerization as a strategy to increase the biological activity of natural and synthetic molecules has been largely pursued in the last several years.^12^ In this context, the binding of a bifunctional ligand to the catalytic sites of an ADAM8 active homodimer on the tumor cell membrane could efficiently reduce ADAM8-mediated tumor growth and invasion to the surrounding tissues. Considering the high homology between the catalytic sites of ADAM8 and ADAM17,^13^ we hypothesized that a bifunctional molecule deriving from the symmetric dimerization of a potent ADAM17 inhibitor could have high activity on ADAM8. In particular, we chose to dimerize the arylsulfonamido-based hydroxamic acid JG26,^14^ (Figure 1) which has already been reported as a nanomolar ADAM17 inhibitor with a good selectivity over MMPs and ADAM10.

**Figure 1:**
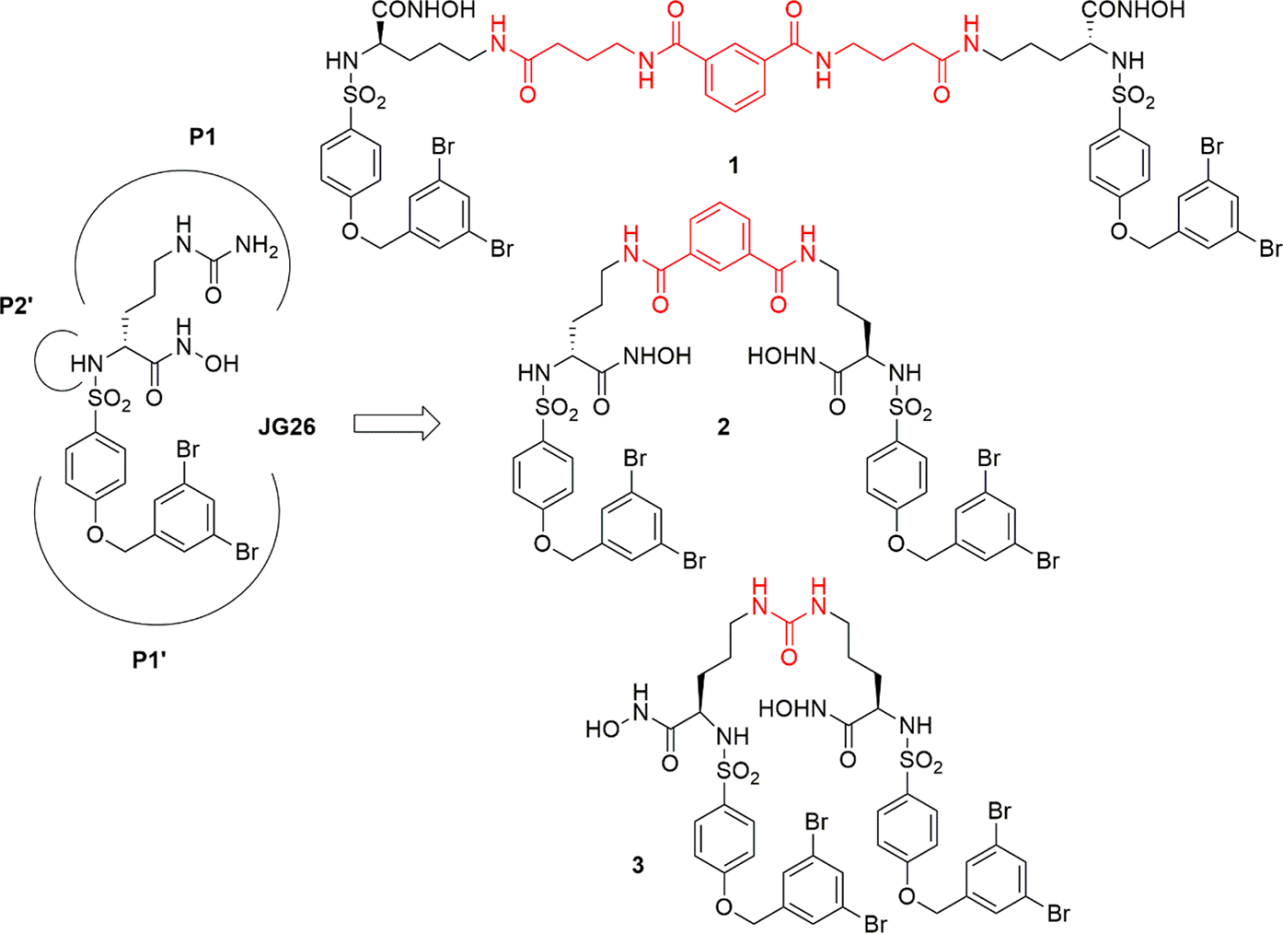
Chemical structure of JG26 and its dimeric derivatives **1**–**3**. The spacer is depicted in red.

As a first step, JG26 was docked in the ADAM8 catalytic domain (cd), and the best docking pose was subjected to 550 ns of molecular dynamics (MD) simulation. As shown in Figure 2, the hydroxamic acid group of the ligand formed a H bond with G302 and E335 and was able to chelate the zinc ion that showed a trigonal bipyramidal chelation geometry. One of the two sulfonamide oxygen atoms showed a H bond with the nitrogen backbone of V301 and G302, whereas the 3,5-dibromobiphenyl moiety was inserted in the S1′ cavity of the enzyme, where it showed lipophilic interactions with I368, F372, and P373. With regards to the ureido portion of the ligand, it did not show stable interactions and was solvent-exposed.

**Figure 2:**
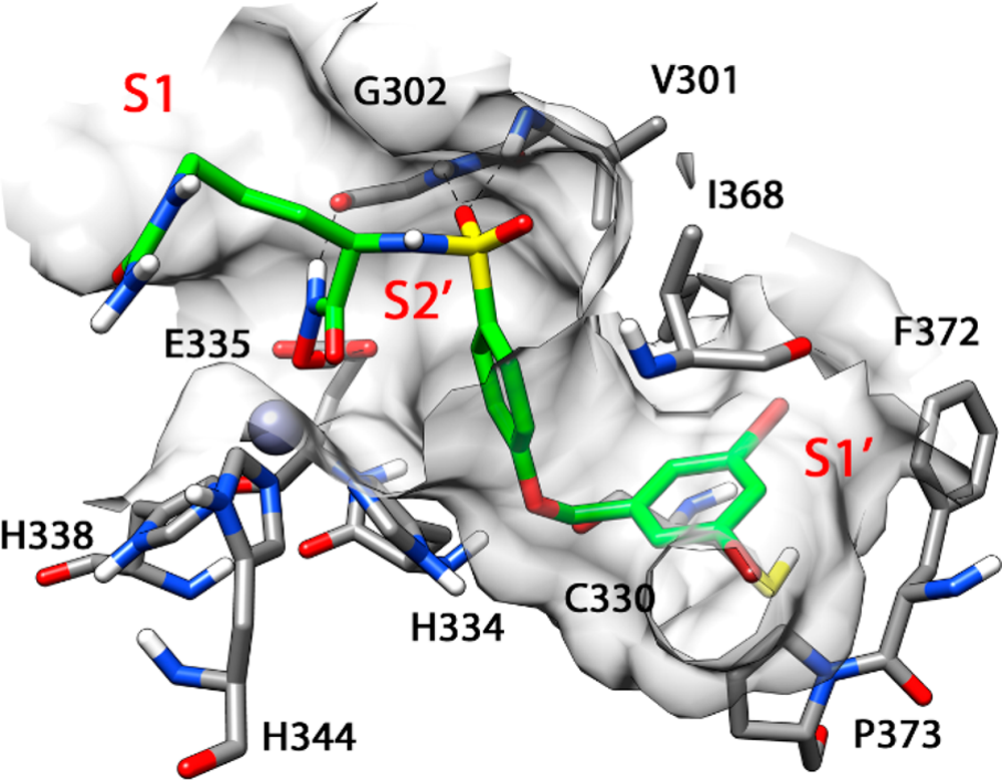
Binding interactions of JG26 with ADAM8 catalytic domain.

Considering the good fitting in ADAM8, JG26 was dimerized in the P1 position through the insertion of a *N*,*N′*-bis(carbamoyl-propyl)-isophthalamide spacer to give compound **1**. The same strategy was successfully applied in the past several years toward the synthesis of bifunctional inhibitors of MT1-MMP with the aim to obtain potent compounds endowed with a reduced cell toxicity with respect to their corresponding monomeric analogues.^15,16^

Then, the crystal structure of the MMP-12 catalytic domain in complex with **1** was solved at 1.23 Å resolution (PDB accession code: 7OVY) with good crystallographic statistics (Supporting Information Table S1) to have a confirmation of the binding interaction of the 3,5-dibromobiphenyl moiety inside the S1′ cavity and to provide hints about a possible arrangement of the trimeric adduct (metalloenzyme–ligand–metalloenzyme). MMP-12 was selected as the metalloenzyme because of the availability of that proteinase in our lab in suitable amounts to perform a crystallographic study according to a previously published protocol^17,18^ and based on the high affinity of compound **1** for this enzyme (IC_50_ = 242 nM, Table 1). Crystallographic analysis showed that the crystal of MMP-12 in complex with compound **1** belongs to space group *C*2 (cell parameters *a* = 51.291, *b* = 60.39, *c* = 54.00; β = 115.23°) (Supporting Information Table S1). The bifunctional inhibitor **1** (assigned occupancy 0.5) crosses the crystallographic two-fold axis of the MMP-12 homodimer, generating a trimeric adduct: MMP-12–**1**–MMP-12 (Supporting Information Figure S1). In detail, the two hydroxamic acid groups of compound **1** chelate the two zinc ions of the MMP-12 homodimer, thus orienting the 3,5-dibromobiphenyl groups into the S1′ cavities.

**Table 1:** *In Vitro* Inhibitory Activity (IC_50_, nM) of New Dimers **1**–**3** and the Monomer JG26 on ADAMs and MMPs^a^

compd	ADAM8	ADAM17	ADAM10	MMP-1	MMP-2	MMP-9	MMP-12	MMP-14
**1**	41	52	30600	415000	4660	3320	242	57000
**2**	73	24	10000	>200000	3000	8800	41	50000
**3**	567	55	27400	348000	900	13500		234000
**JG26**	12	1.9	150	>500000	240	1630	9.4	19500

a Enzymatic data are mean values for three independent experiments performed in duplicate. Standard deviations (SD)s were generally within ±10%.

As reported above, this X-ray structure confirmed the binding interaction of the 3,5-dibromobiphenyl moiety inside the S1′ cavity and provided a potential disposition of compound **1** for interacting with two metalloenzymes. To verify if this arrangement could also be plausible for the ADAM8–**1**–ADAM8 trimer, we aligned two ADAM8–JG26 complexes on the MMP-12–**1**–MMP-12 adduct, maintaining the same *N*,*N*′-bis(carbamoyl-propyl)-isophthalamide spacer present in **1**. The obtained complex was then subjected to 1.051 ms of MD simulation to analyze the stability of the trimeric complex. As shown in Figure S2, after ∼500 ns of MD simulation, the system reached a good stability, with the α-carbons of the two proteins showing an rmsd of ∼3.5 Å and the heavy atoms of compound **1** with an rmsd of ∼5.0 Å, thus confirming a good match between the experimental MMP-12–**1**–MMP-12 and the theoretical ADAM8–**1**–ADAM8 3D disposition. Figure 3A shows the average structure of the ADAM8–**1**–ADAM8 trimeric system; the ligand kept the two monomers very close to each other, and there was also a H-bond network between the fragment H259–T267 of one monomer (monomer A) and the fragment H344–Q349 of the other monomer (monomer B). As shown in Figure 3B, the catalytic H344A formed a H bond with D263B, which also showed an interaction with G366A; N347A formed a H bond with E269B, and E346A formed H bonds with H259B and S261B, whereas Q349A formed a H bond with T273B.

**Figure 3:**
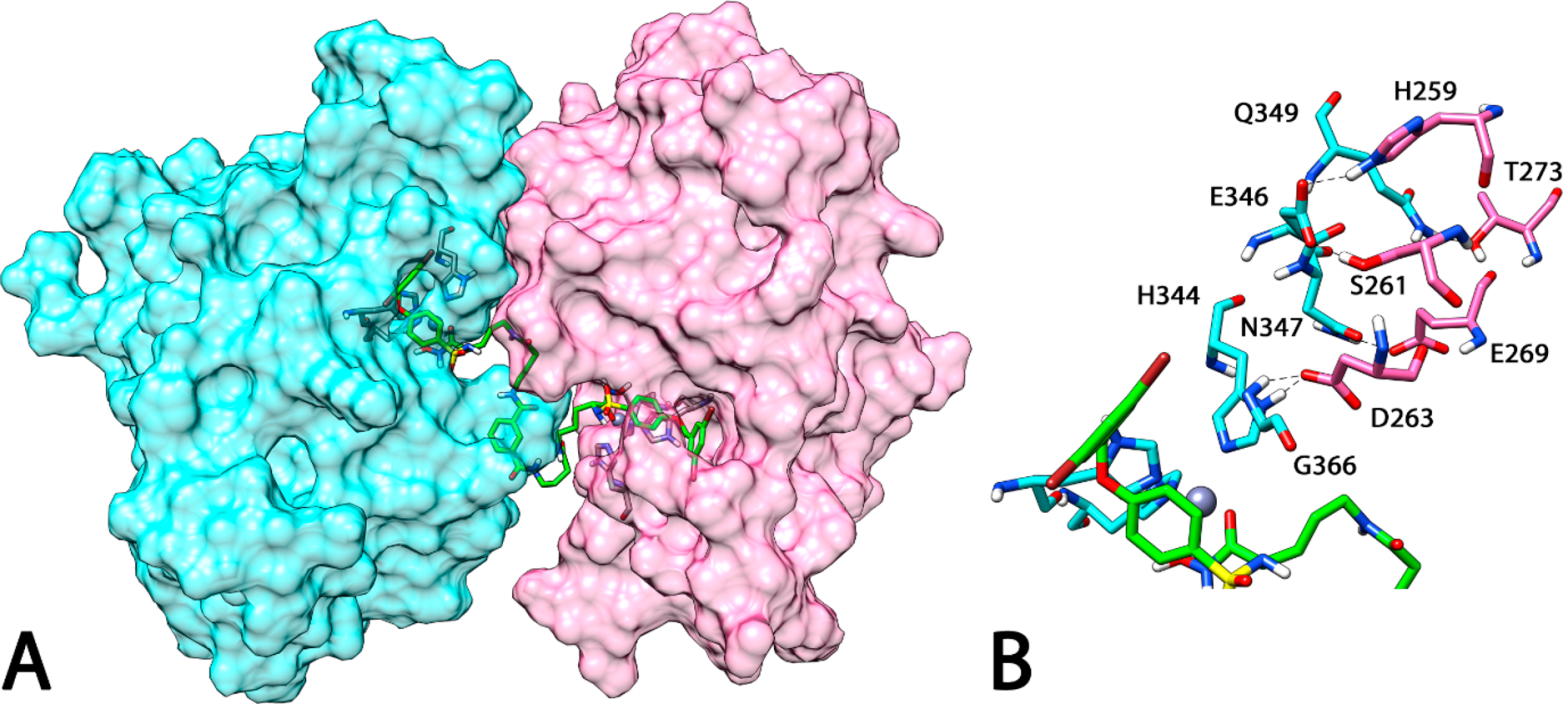
(A) Proposed trimeric adduct ADAM8–**1**–ADAM8. (B) H-bond network between the two ADAM8 monomers.

Because the computational studies validated our hypothesis to use a bivalent compound to inhibit a dimeric form of ADAM8, we synthesized two other symmetric derivatives of JG26, compounds **2** and **3** (Figure 1), respectively, linked through an isophthalamide and an ureido spacer, to prove the effect of the linker length on the inhibitory potency and selectivity.

The new compounds were first tested *in vitro* on recombinant ADAM8 in comparison with JG26; then, cell-based assays were performed to determine the effect of these compounds in a more complex system. The inhibition of CD23 cleavage and the release of its soluble form from HEK293 cells was used to evaluate their ability to inhibit the proteolytic activity of ADAM8. For further functional characterization, invasion assays were conducted on breast cancer cells MDA-MB-231.

Compounds **1** and **2** were synthesized as reported in Scheme 1. Hydroxamic acid **4** was prepared as previously described.^19^ The di-NHS (*N*-hydroxysuccinimido)-activated esters **5**(15) and **6**(20) were reacted with amine **4** in Dimethyl sulfoxide (DMSO) in the presence of *N*,*N*-diisopropylethylamine (DIPEA) to afford, respectively, the dimeric hydroxamates **1** and **2** after chromatographic purification.

**Scheme 1:**
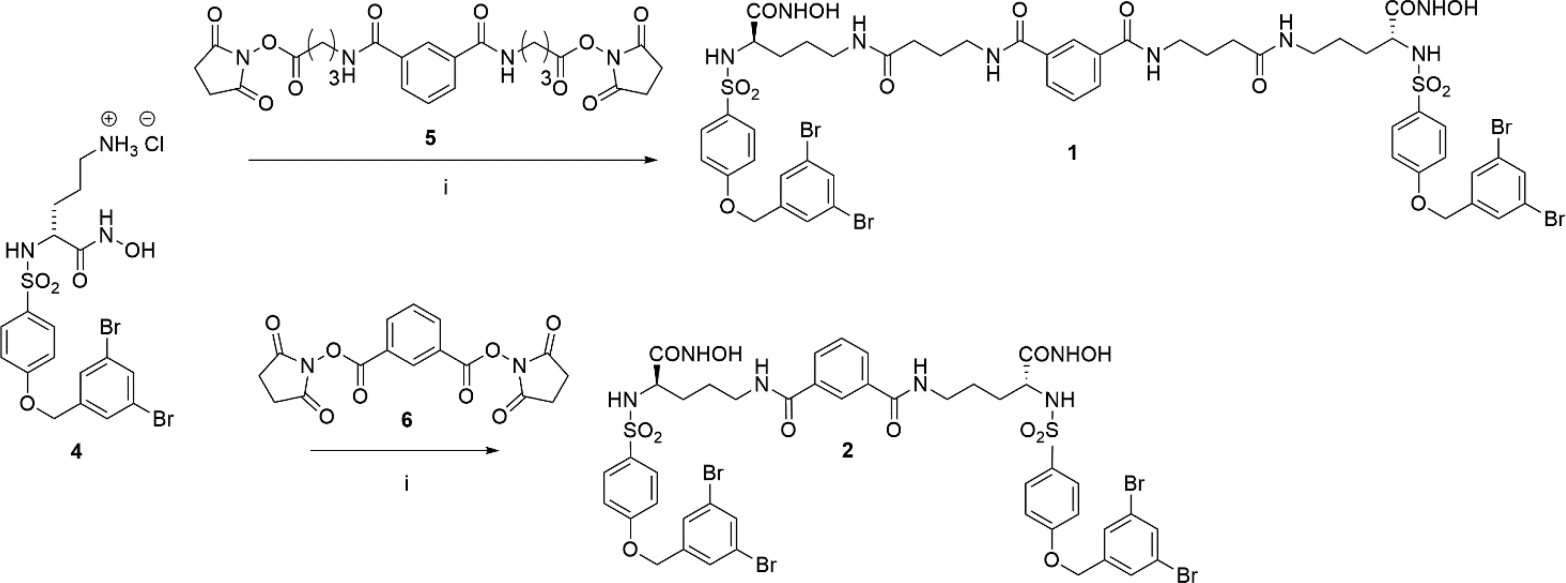
Synthesis of Compounds **1** and **2** Reagents and conditions: (i) DIPEA, DMSO, rt, 24 h.

Compound **3** was synthesized as reported in Scheme 2. The already reported carboxylic acid **7**(19) was protected as *tert*-butyl ester by the reaction with *N*,*N*-dimethylformamide-di-*tert*-butyl acetal at 95 °C. The amine **9** was obtained as a trifluoroacetate salt by the selective hydrolysis of the Boc group in the presence of the *tert*-butyl group upon the treatment of compound **8** with trifluoroacetic acid (TFA) under controlled conditions. The free amine **10** was then obtained by the treatment of **9** with NaHCO_3_ for 30 min. The condensation between two units of **10** was obtained using 1,1′-carbonyldiimidazole (CDI) as a condensation agent to afford the dimer **11**. *tert*-Butyl ester hydrolysis with TFA gave carboxylic acid **12**. This dicarboxylate was finally converted into the corresponding dihydroxamate **3** by condensation with *O*-(tetrahydropyranyl)hydroxylamine (THP-ONH_2_) followed by treatment with TFA.

**Scheme 2:**
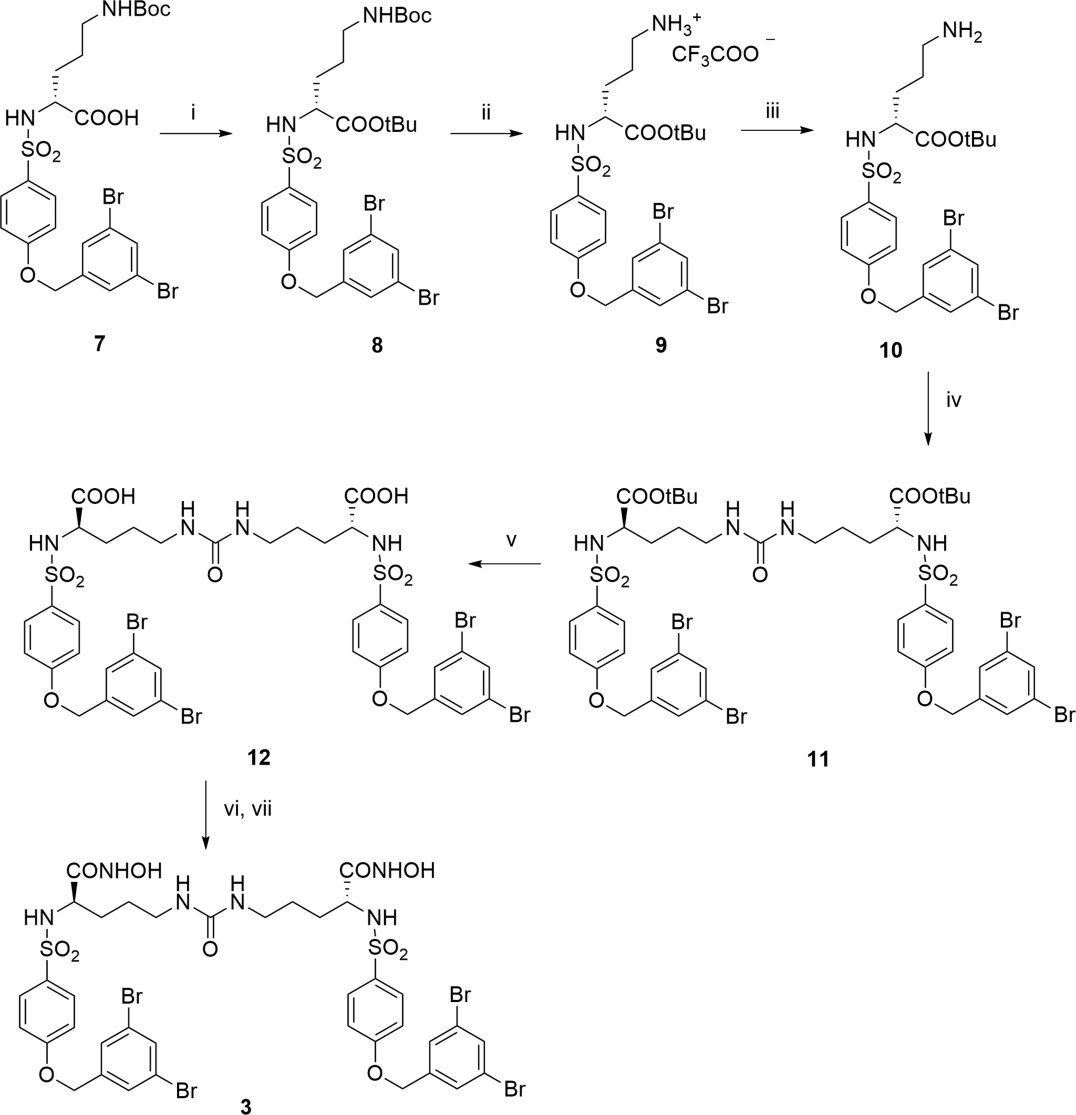
Synthesis of Compound **3** Reagents and conditions: (i) *N*,*N*-DMF-di-*tert*-butyl acetal, toluene, 105 °C; (ii) TFA, DCM, 1 h, 0 °C; (iii) NaHCO_3_, CHCl_3_, rt; (iv) CDI, DCM, rt, 18 h; (v) TFA, DCM, 0 °C to rt; (vi) THP-ONH_2_, HOBt, EDC, NMM, DMF, rt; (vii) TFA, DCM, rt.

The newly synthesized bivalent derivatives **1**–**3** were then tested *in vitro* on human recombinant ADAM8 in comparison with JG26 by a fluorometric assay. The inhibitory activity against a panel of MMPs and ADAM10/17 was determined as well to assess their selectivity profile. Data are reported in Table 1 as IC_50_ values (nM).

All new bifunctional JG26 derivatives showed a nanomolar activity against ADAM8 that was only slightly lower than that of their parent compound (IC_50_ = 12 nM). However, the insertion of a shorter linker between the two warheads caused a drop in activity, as shown by ureido derivative **3** (IC_50_ = 567 nM). In fact, the last one was 14 times less active than compound **1** on ADAM8. With regard to their selectivity profile, all compounds were good inhibitors of ADAM17 but showed a high selectivity over ADAM10 and most of the tested MMPs. In particular, compound **1** had a 740-fold selectivity for ADAM8 over ADAM10. Next, these compounds were tested for their ability to inhibit the shedding of an ADAM8-dependent cell surface protein, the low affinity IgE receptor CD23, *in vivo*. In brief, a human embryonic kidney (HEK) cell line expressing ADAM8 and CD23^13^ was incubated with different concentrations of inhibitors, and the release of soluble CD23 in the cell supernatants was determined by a CD23 enzyme-linked immunosorbent assay (ELISA) (Figure 4).

**Figure 4:**
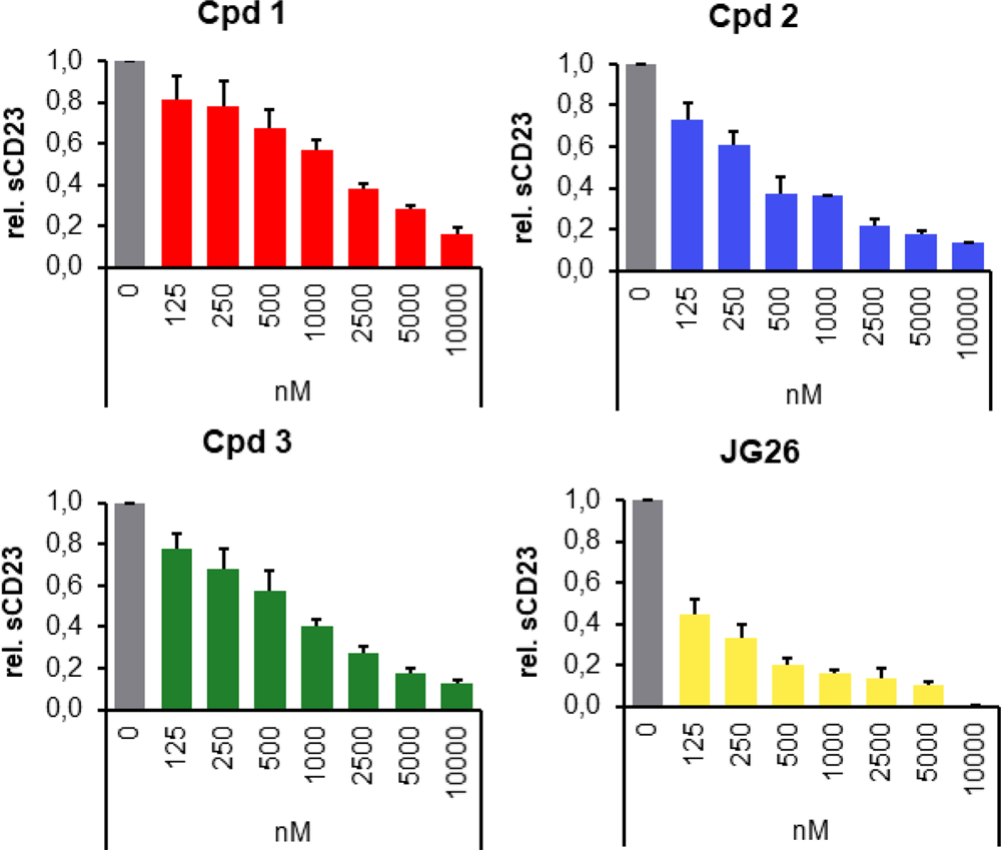
Effects of new compounds **1**–**3** and JG26 (125 nM to 10 μM) on the shedding of CD23 in a cell-based assay using double-stable HEK cells (ADAM8/CD23). After the incubation of cells with **1**–**3** and JG26 for 24 h, cell supernatants were collected and subjected to an ELISA to determine the amount of soluble CD23. Data are the mean values from three independent experiments performed in triplicate. The following IC_50_ values were calculated: Cpd **1**: >1500 nM; Cpd **2**: 350 nM; Cpd **3**: 800 nM; JG26: 120 nM.

Except for compound **1**, the IC_50_ values for the cell-based activities were in agreement with those observed in the *in vitro* enzyme assays for ADAM8. Thus JG26 and compound **2** were the most potent inhibitors for ADAM8 in this assay. With the aim of rationalizing these cell-based activity results, the main physical–chemical properties of compounds **1**–**3** were *in silico* investigated by using the SwissADME tools.^21^ The octanol/water partition coefficient (expressed as logP), which is commonly used as an indicator of the molecular lipophilicity, and the aqueous solubility (expressed as logS) of the compounds were predicted using a consensus strategy, that is, combining the five logP and the three logS calculation methods, respectively, available in the web tool. As shown in Table S2, all three compounds show a high predicted logP value (>4.5) and a low water logS solubility (lower than −11); furthermore, there are no marked differences between the three compounds or, in particular, between compound **1** and **2**, thus suggesting that the inactivity of compound **1** should not be ascribed to these factors.

JG26 and compounds **1**–**3** were finally tested (Figure 5) for their capacity to affect the invasive potential of tumor cells, an effect that, at least in part, is dependent on the ADAM8 proteolytic activity, as ADAM8-deficient cell lines suggest.^5,7^

**Figure 5:**
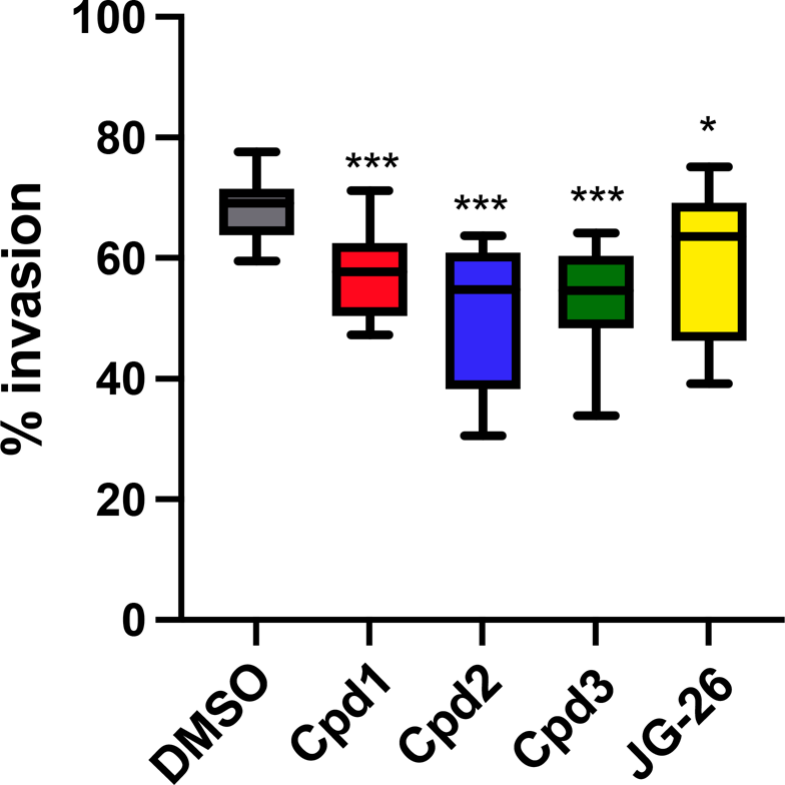
Effects of compounds **1**–**3** and JG26 on cancer cell invasion. MDA-MB-231 cells were pretreated with inhibitors for 24 h prior to the assay. Boxplot of invaded cells in %. Results are obtained from three independent experiments with counting of five randomly chosen viewing fields. One-way ANOVA was performed to determine statistical significance with * *p* < 0.05 and *** *p* < 0.001.

Significant effects on invasion were observed for compounds **1** (57.25 ± 7.24%, *p* < 0.001), **2** (50.8 ± 11.4%, *p* < 0.001), and **3** (52.9 ± 9.14%, *p* < 0.001), whereas JG26 had the lowest effect on cell invasion (59.87 ± 12.27%, *p* < 0.05) compared with the control (69.07 ± 5.26%). Because JG26 is the most potent inhibitor of ADAM8 protease activity (Table 1 and Figure 4), we conclude that compounds **1**–**3** act primarily through protease inhibition and possibly do not interact only with the catalytic site due to their elongated structure. The hypothesis that they could interfere with the ADAM8 disintegrin domain signaling has been proven by Western blots for phosphorylated Akt and ERK1/2, which were unaffected by the compounds (Supporting Information Figure S3), so that ADAM8-dependent intracellular signaling was not addressed.

In summary, new bifunctional ADAM8 inhibitors selective over ADAM10 and several MMPs involved in tumor progression have been developed. Following a dimerization strategy, a first bifunctional derivative has been synthesized starting from an arylsulfonamido-based hydroxamic acid, JG26, previously reported as an ADAM17 inhibitor. The dimeric compound **1** was obtained through the insertion of an *N*,*N*′-bis(carbamoyl-propyl)-isophthalamide symmetric linker. X-ray crystallographic analysis in MMP-12 cd taken as model metalloprotease and molecular modeling studies in ADAM8 cd validated the design of **1**, driving the synthesis of two other analogues bearing linkers of different lengths. All JG26 dimeric derivatives were tested *in vitro* and *in vivo* for their ability to block ADAM8 proteolytic activity in comparison with their parent compound. Compound **2**, bearing an isophthalamide linker of intermediate length, showed good inhibitory results both on ADAM8 and on CD23 shedding in HEK cells. Moreover, this derivative was the most active in inhibiting the invasiveness of MDA-MB-231 breast cancer cells, even compared with JG26. These preliminary results proved the efficacy of bifunctional inhibitors in cancer cells overexpressing ADAM8, but further studies will be needed to improve the selectivity of compound **2** for ADAM8 over ADAM17 and to ultimately define its mechanism of action at a molecular level. Furthermore, thanks to the proposed molecular modeling procedure herein reported, further theoretical studies will be developed for investigating the role of the linker length in terms of the ADAM8 activity and the selectivity toward the other metalloenzymes.

## Supporting Information Available

Supplementary figures, experimental details, crystallographic data, and compound characterization (PDF)

## Accession Codes

The crystal structure for the complex between 1 and MMP-12 has been deposited at the Protein Database (PDB) with the code 7OVY.

## Abbreviations

ADAM: a disintegrin and metalloproteinase
MMP: matrix metalloproteinase
EGF: epidermal growth factor
ECM: extracellular matrix
MD: molecular dynamics
rmsd: root-mean-square deviation
MT1-MMP: membrane type 1 matrix metalloproteinase
DIPEA: *N*,*N*-diisopropylethylamine
EDC: *N*-(3-(dimethylamino)propyl)-*N*′-ethylcarbodiimide hydrochloride
NMM: *N*-methylmorpholine
CDI: 1,1′-carbonyldiimidazole
TFA: trifluoroacetic acid
HOBt: 1-hydroxybenzotriazole
THP: *O*-(tetrahydropyranyl)
NHS: (*N*-hydroxysuccinimido)
ELISA: enzyme-linked immunosorbent assay
