# Supplementary material for: Discovery of Dimeric Arylsulfonamides as Potent ADAM8 Inhibitors
Source: ACS Med Chem Lett. 2021 Oct 8;12(11):1787–93. doi: 10.1021/acsmedchemlett.1c00411 (PMC8805605; doi:10.1021/acsmedchemlett.1c00411)
Supplement: Supplementary file 1 — ml1c00411_si_001.pdf [file ml1c00411_si_001.pdf]

SUPPORTING INFORMATION FOR

Discovery of dimeric arylsulfonamides as potent ADAM8 Inhibitors

Doretta Cuffaro,[1] Caterina Camodeca,[1] Tiziano Tuccinardi,[1] Lidia Ciccone,[1] Jörg W. Bartsch,[2] Tanja Kellermann,[2] Lena Cook,[2] Elisa Nuti,[1,] and Armando Rossello[1]*

[1] Department of Pharmacy, University of Pisa, via Bonanno 6, 56126 Pisa, Italy.

[2] Department of Neurosurgery, Marburg University, Baldingerstrasse, 35033 Marburg, Germany

*Corresponding author: Elisa Nuti, phone, +39 050 2219551, e-mail: elisa.nuti@unipi.it

Table of Contents

I. Supplementary figures S2

II. General synthetic methods S4

III. Synthetic details and characterization of compounds S4

A. Synthesis of 1-2 (details for Scheme 1) S4

B. Synthesis of 3 (details for Scheme 2) S6

IV. Crystallization and structure resolution S8

V. Molecular Modeling Studies S9

VI. Biological Activity on Isolated Enzymes S11

VII. Biological Activity on cells S12

VIII. Representative NMR spectra S14

IX. References S19

I. Supplementary figures

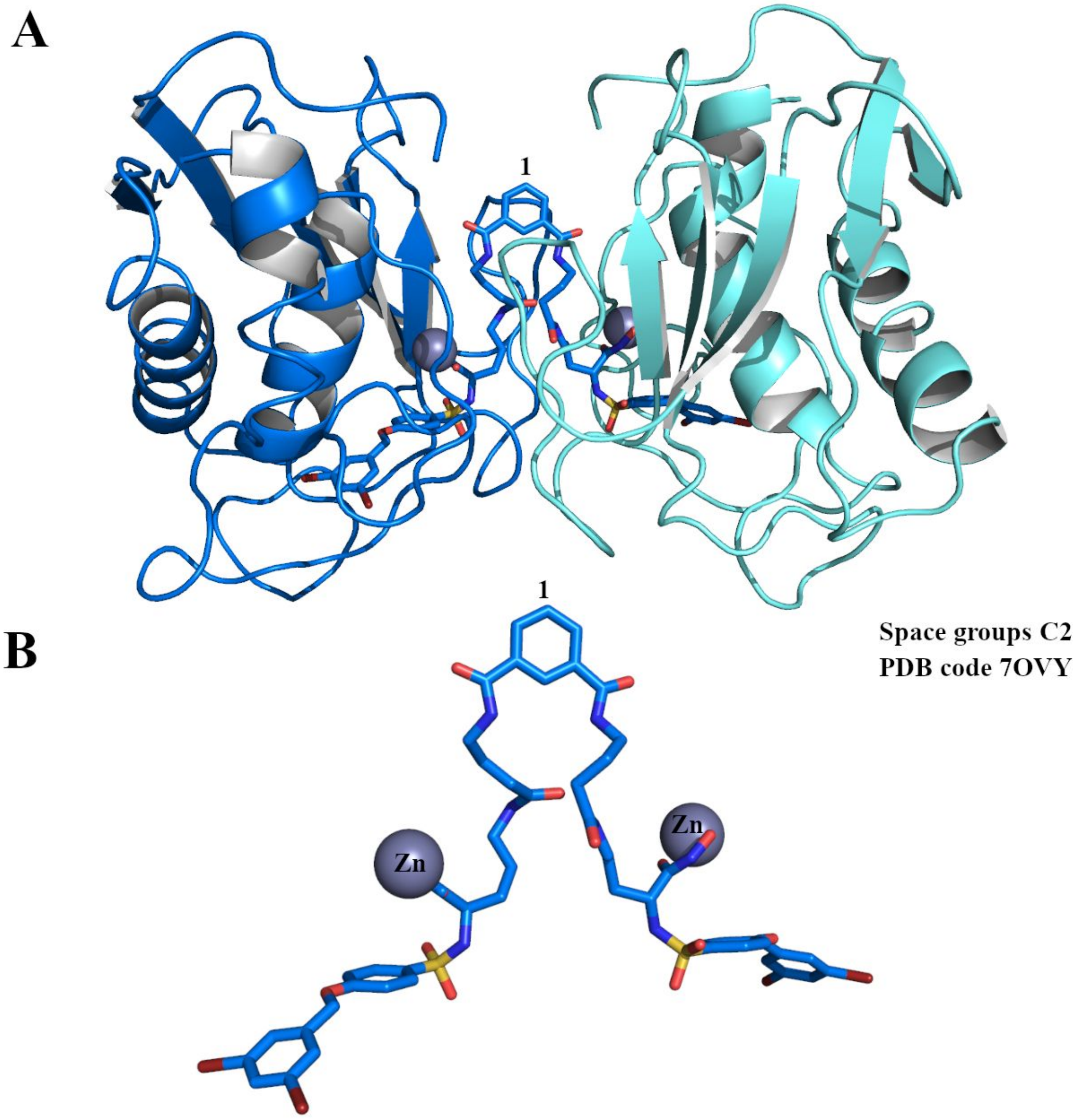

Figure S1. Graphical representation of MMP-12 in complex with compound **1**. (A) X-ray crystal structure of homodimer MMP-12 in complex with inhibitor **1** (PDB 7OVY); (B) Zoom of the ligand: the two hydroxamic acid groups of the compound **1** chelate the two zinc ions of the MMP-12 homodimer.

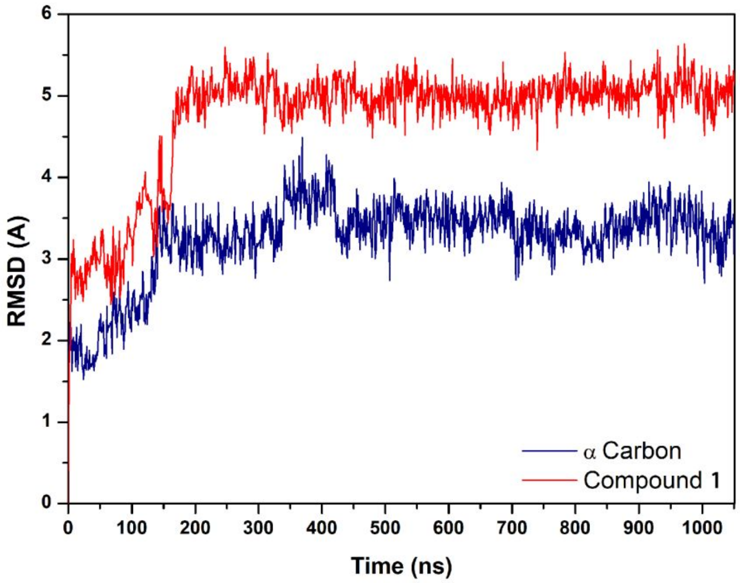

Figure S2. Analysis of the MD simulation of the ADAM8-**1**-ADAM8 trimeric adduct. RMSD in angstrom of the α carbon of the protein (blue line) and ligand heavy atoms (red line) from the starting model structure during the simulation.

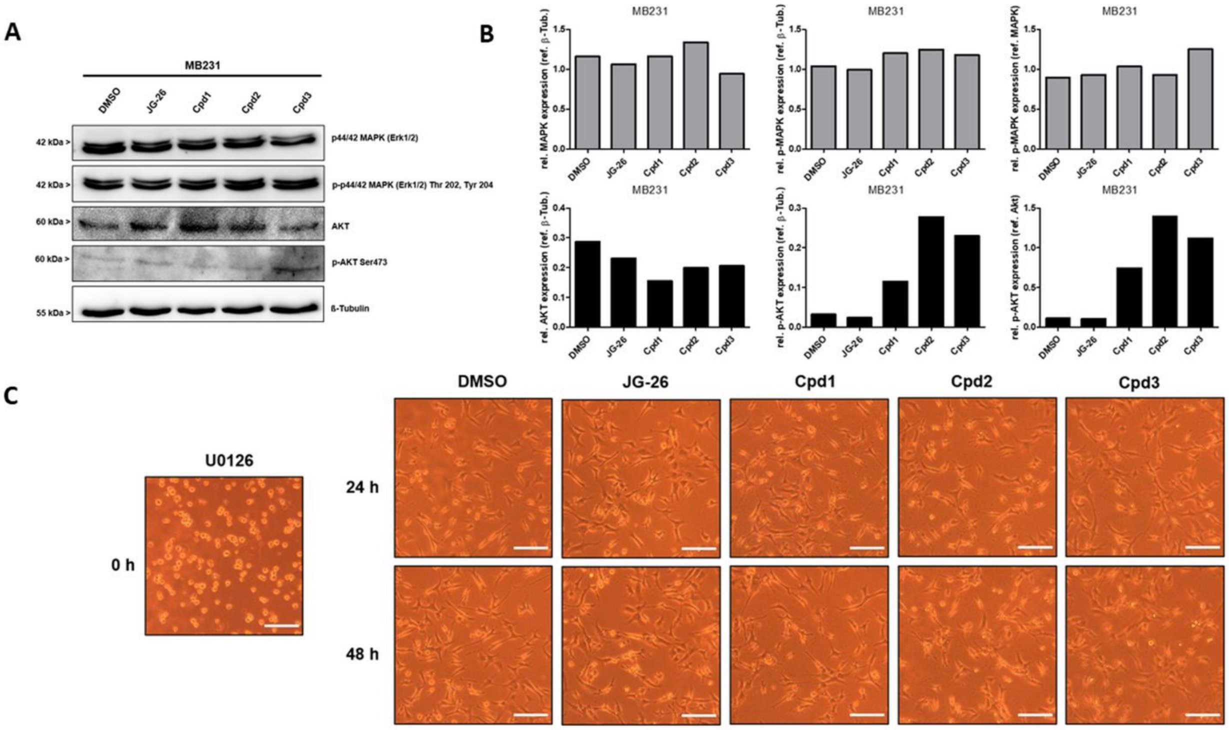

Figure S3. Intracellular kinase signaling after treatment of MB231 with JG-26, Cpd**1**, Cpd**2** or Cpd**3**. (A) Western blot (WB) of kinase phosphorylation of the MAPK and AKT signaling pathways in MB231 cells after 48 h incubation with either DMSO as control, JG-26 (10 μM), Cpd1 (10 μM), Cpd2 (10 μM) or Cpd3 (10 μM). Before incubation, cells were pretreated with MAPK inhibitor U0126 (10 μM) for 24 h. (B) Relative protein expression of MAPK (referred to ß-Tubulin), p-MAPK (referred to ß-Tubulin) and p-MAPK (referred to MAPK) are shown in the upper and AKT (referred to ß-Tubulin), p-AKT (referred to ß-Tubulin) and p-AKT (referred to AKT) in the lower row. (C) Morphology of MB231 cells after pretreatment with U0126 (left, 0h) and after incubation with JG-26, Cpd1, Cpd2, Cpd3 (right, upper and lower panel, 24 h and 48 h). Scale bar, 150 μm.

II. General synthetic methods

Melting points were determined on a Leica Galen III Microscope (Leica/Cambridge Instruments) and are uncorrected. ^{1}H and ^{13}C NMR spectra were recorded on a Bruker Avance III HD 400 MHz spectrometer. Chemical shifts (δ) are reported in parts per million and coupling constants (*J*) are reported in hertz (Hz). ^{13}C NMR spectra were fully decoupled. The following abbreviations were used to explain molteplicities: singlet (s), doublet (d), triplet (t), double doublet (dd), broad (br), and multiplet (m). Chromatographic separations were performed on silica gel columns by flash column chromatography (Kieselgel 40, 0.040−0.063 mm, Merck) or using ISOLUTE Flash Si II cartridges (Biotage). Reactions were followed by thin-layer chromatography (TLC) on Merck aluminum silica gel (60 F254) sheets that were visualized under a UV lamp, and hydroxamic acids were visualized with $FeCl_3$ aqueous solution. Evaporation was performed in vacuo (rotating evaporator). Sodium sulfate was always used as the drying agent. Commercially available chemicals were purchased from Sigma-Aldrich. Elemental analysis was used to determine the purity of target compounds. Analytical results are within ± 0.4% of the theoretical values. The ESI-MS spectra were recorded by direct injection at 5 (positive) and 7 (negative) μL min^{-1} flow rate in an Orbitrap high-resolution mass spectrometer (Thermo, San Jose, CA, USA), equipped with HESI source.

III. Synthetic details and characterization of compounds

Scheme 1

General procedure for the synthesis of dimers 1 and 2

To a solution of the hydroxamic acid **4** (2 eq) in DMSO, DIPEA (2 eq) and the proper di-NHS ester **5** or **6** (1 eq) were added dropwise. The reaction mixture was stirred at room temperature for 24h, under argon atmosphere. Then Et_2O was added to the reaction mixture and cooled to 0°C. The solvent was removed by decantation and the residual oil was evaporated. The crude was purified by reverse phase flash chromatography (C18) to afford the pure compound as a white solid.

*N^1,N^3-bis(4-(((R)-4-(4-((3,5-dibromobenzyl)oxy)phenylsulfonamido)-5-(hydroxyamino)-5-oxopentyl)amino)-4-oxobutyl)isophthalamide (**1**)*

Compound **4** (250 mg, 0.38 mmol) was dissolved in DMSO (1 mL), and then di-NHS ester **5** (100 mg, 0.19 mmol) and Et_3N (0.16 mL, 1.13 mmol), were added. The dimer **1** was synthesized following the general procedure and then the crude was purified by C18 flash chromatography using an ISOLUTE C18 5g ((MeOH+CH_3CN (1:1)) 7 : (H_2O/CH_3COONH_4 (1 g/L)) 3) affording compound **1** as a white solid (11 mg, 4% yield). Mp 122-125 °C. ^{1}H NMR (400 MHz, CD_3OD) δ: 1.40-1.75 (m, 8H); 1.87-1.94 (m, 4H); 2.20-2.33 (m, 4H); 3.05- 3.18 (m, 4H); 3.42 (t, J = 6.6 Hz, 4H); 3.52-3.73 (m, 2H); 5.17 (s, 4H); 7.11-7.14 (m, 4H); 7.58-7.64 (m, 5H); 7.70 (bs, 2H); 7.77-7.98 (m, 4H); 8.34 (s, 1H). ^{13}C NMR (CD_3OD) δ: 19.4; 26.5; 32.0; 34.5; 39.6; 40.6; 55.5; 69.6; 103.2; 116.2; 123.9; 127.2; 130.2; 131.1; 134.4; 136.1; 142.3; 162.9; 169.3; 170.1; 175.4. Elemental analysis calcd (%) for $C_{52}H_{58}Br_4N_8O_{14}S_2$: C 44.52, H 4.17, N 7.99; found: C 44.50, H 4.20, N 8.00.

*Synthesis of N^1,N^3-bis((R)-4-(4-((3,5-dibromobenzyl)oxy)phenylsulfonamido)-5-(hydroxyamino)-5-oxopentyl)isophthalamide **(2).***

Compound **4** (320 mg, 0.54 mmol) was dissolved in DMSO (7 mL), and then di-NHS ester **6** (100 mg, 0.27 mmol) and DIPEA (0.20 mL, 1.08 mmol) were added dropwise. The dimer **2** was synthesized following the general procedure and then the crude product was purified by C18 flash chromatography using a ISOLUTE C18 5g (CH_3CN/MeOH/H_2O (5:1:4)) to afford compound **2** as a white solid (63 mg, 19% yield). M.p. 48-49 °C. ^{1}H NMR (400 MHz, CD_3OD) δ: 1.42-1.73 (m, 8H), 3.22-3.30 (m, 4H), 3.66-3.69 (m, 2H), 5.09 (s, 4H), 7.12 (d, J =9.0 Hz, 4H), 7.50-7.54 (m, 1H), 7.58 (d, J =1.8 Hz, 4H), 7.70 (t, J =1.8 Hz, 2H), 7.82 (d, J =9.0 Hz, 4H), 7.92 (dd, J_1= 7.8 Hz, J_2= 1.70 Hz, 2H), 8.23-8.25 (m, 1H). ^{13}C NMR (100 MHz, CD_3OD) δ: 25.1, 30.1, 38.8, 54.0, 68.0, 114.7, 122.6, 125.8, 128.4, 128.8, 128.9, 129.7, 132.8, 133.0, 134.7, 140.9, 161.5, 167.9, 168.9. Elemental analysis calcd (%) for $C_{44}H_{44}Br_4N_6O_{12}S_2$: C 42.87, H 3.60, N 6.82; found: C 42.90, H 3.62, N 6.80. HRMS (ESI) m/z

calculated for $[C_{44}H_{44}Br_4S_2N_6O_{12}\text{-}H]^-$: 1226.9119, found: 1226.9082; calculated for $[C_{44}H_{44}Br_4S_2N_6O_{12}+Cl]^-$: 1262.8886, found 1262.8887.

Scheme 2

*(R)-tert-Butyl 5-((tert-butoxycarbonyl)amino)-2-(4-((3,5-dibromobenzyl)oxy)phenylsulfonamido)pentanoate (**8**)*

To a solution of **7** (500 mg, 0.78 mmol) in dry toluene (1.55 mL), DMF-di-*tert*-butyl acetal (0.75 mL) was added dropwise under inert atmosphere (Ar). The reaction mixture was stirred for 2h at 105 °C, then the solvent was evaporated. The crude (700 mg) was purified by flash chromatography using prepacked Isolute column Si II 20g (*n*-hexane /EtOAc in gradient from 3:1 to 1:1) affording compound **8** pure as a white solid (324 mg, 60% yield). ^{1}H NMR (400 MHz, DMSO-d_6) δ: 1.17 (s, 9H); 1.36 (s, 9H); 1.53-1.51 (m, 4H); 2.84 (m, 2H); 3.56 (m, 1H); 5.21 (s, 2H); 6.80 (br t, *J*= 6 Hz, 1H); 7.16 (m, 2H); 7.69-7.68 (m, 4H); 7.83 (t, *J*= 2 Hz, 1H); 8.06 (d, *J*= 8.8 Hz, 1H).

*(R)-5-(tert-Butoxy)-4-(4-((3,5-dibromobenzyl)oxy)phenylsulfonamido)-5-oxopentan-1-amine trifluoro acetate salt (**9**)*

Trifluoroacetic acid (2.05 mL, 26.79 mmol) was added dropwise to a stirred solution of *tert*-butyl ester **8** (324 mg, 0.47 mmol) in dry DCM (9.82 mL), cooled to 0 °C. The solution was stirred for 1 h at 0 °C. The solvent was then removed in vacuo and co-evaporated twice with DCM. The crude was purified by flash chromatography using a Isolute cartridge Si II 10 g ($CHCl_3$: MeOH 20:1) to give the carboxylic acid **9** as a white solid (172 mg, 52% yield). ^{1}H NMR (400 MHz, DMSO-d_6) δ: 1.15 (s, 9H); 1.67-1.50 (m, 4H); 2.76 (m, 2H); 3.62 (m, 1H); 5.22 (s, 2H); 7.16 (m, 2H); 7.71-7.68 (m, 4H); 7.83 (t, *J*= 2 Hz, 1H); ^{19}F NMR (376 MHz, DMSO-d_6) δ: -73.4.

*(R)-5-(tert-Butoxy)-4-(4-((3,5-dibromobenzyl)oxy)phenylsulfonamido)-5-oxopentan-1-amine (**10**)*

To a solution of **9** (172 mg, 0.24 mmol) in 2 mL of $CHCl_3$, a saturated solution of $NaHCO_3$ (3mL) was added. The mixture was stirred for 1h a rt, then $CHCl_3$ was added and the resulting mixture was washed with water. The organic phase was dried with Na_2SO_4, filtered and evaporated. The crude was purified by flash chromatography using a Isolute cartridge Si II 10 g ($CHCl_3$: MeOH 20:1) to give the free amine **10** (125 mg, 88%yield) as a white solid. ^{1}H NMR (400 MHz, DMSO-d_6) δ: 1.18 (s, 9H); 1.62-1.44 (m, 4H); 2.52 (m, 2H); 3.56 (m, 1H); 5.21 (s, 2H); 7.16 (m, 2H); 7.70-7.68 (m, 4H); 7.81 (m, 1H).

*di-tert-Butyl 5,5'-(carbonylbis(azanediyl))bis(2-(4-((3,5-dibromobenzyl)oxy)phenylsulfonamido)pentanoate) (**11**)*

To a solution of the amine **10** (195 mg, 0.329 mmol) in 5.13 mL of DCM, carbonyldimidazole (CDI) (324 mg, 0.329 mmol) and *N*-methylmorpholine (18 µL) were added. The reaction mixture was stirred at rt overnight and then, was solved with DCM and washed with water and brine. The organic phase was dried over Na_2SO_4, filtered and evaporated. The crude was purified by Flash chromatography using a Isolute cartridge Si II 5 g (in gradient from 100% $CHCl_3$ to $CHCl_3$: MeOH 200:1) to give the dimeric ureidic di-*tert* butyl ester **11** as a white solid (115 mg; 58% yield). ^{1}H NMR (400 MHz, DMSO-d_6) δ: 1.16 (s, 18H); 1.21-1.58 (m, 8H); 2.88 (dd, *J*= 6.4 Hz, 4H); 3.56 (m, 2H); 5.20 (s, 4H); 5.76 (t, *J*= 5.6 Hz, 2H); 7.15 (m,4H, Ar); 7.69-7.67 (m, 8H); 7.82 (m, 2H); 8.05 (d, *J*= 8.8 Hz, 2H).

*5,5'-(Carbonylbis(azanediyl))bis(2-(4-((3,5-dibromobenzyl)oxy)phenylsulfonamido)pentanoic acid) (**12**)*

The diester **11** (100 mg, 0.083 mmol) was dissolved in 5 mL of DCM and trifluoroacetic acid (1.45 mL) was added. The solution was stirred for 20h at rt, then the solvent was removed. The crude was co-evaporated (3x) with DCM and the resulting solid was triturated with Et_2O affording the dicarboxylic acid **12** as a white solid (86 mg, 95% yield). ^{1}H NMR (400 MHz, DMSO-d_6) δ: 1.25-1.58 (m, 8H); 2.87 (m, 4H); 3.58-3.64 (m, 2H); 5.18 (s, 4H); 5.74 (t, *J*= 4.8 Hz, 2H); 7.15 (m, 4H, Ar); 7.67-7.72 (m, 8H); 7.83 (m, 2H); 7.99 (d, *J*= 8.8 Hz, 2H). ^{13}C NMR (100 MHz, DMSO-d_6) δ: 26.3; 29.7; 55.4; 67.8; 114.9; 122.6; 128.7; 129.5; 133.0, 133.5; 141.2; 158.0; 160.7; 172.8.

*(2R,2'R)-5,5'-(Carbonylbis(azanediyl))bis(2-(4-((3,5-dibromobenzyl)oxy)phenylsulfonamido)-N-((tetrahydro-2H-pyran-2-yl)oxy)pentanamide) (**13**)*

To a stirred solution of the dicarboxylic acid **12** (77 mg, 0.07 mmol) in dry DMF (0.5 mL) 1-hydroxybenzotriazole (HOBt) (22.7 mg, 0.168 mmol), *O*-(tetrahydro-2H-pyran-2-yl)hydroxylamine ($THPONH_2$) (51 mg, 0.434 mmol), *N*-methylmorpholine (46 µL, 0.42 mmol) and 1-[3-(Dimethylamino)propyl]-3-ethyl carbodiimide hydrochloride (EDC) (38 mg, 0.196 mmol) were added under nitrogen atmosphere. After stirring at rt overnight, the mixture was dissolved in EtOAc and washed with water, $NaHCO_3$ saturated solution and brine. Then the organic phase was dried over Na_2SO_4 and evaporated in vacuum. The crude was triturated with Et_2O affording *O*-tetrahydropyranyl derivative **13** as diastereoisomeric mixture (80 mg, 88% yield). ^{1}H NMR (400 MHz, DMSO-d_6) δ:

1.15-1.60 (m, 20H); 2.83-2.89 (m, 4H); 3.45 (t, *J*= 10.4 Hz, 2H); 3.57-3.63 (m, 2H); 3.85-3.81 (m, 2H); 4.31 (br s, 1H); 4.68 (br s, 1H); 5.19 (s, 4H); 5.79- 5.74 (m, 2H); 7.13 (m, 4H, Ar); 7.67-7.71 (m, 8H); 7.83 (m, 2H); 7.89-7.96 (m,2H).

*(2R,2'R)-5,5'-(Carbonylbis(azanediyl))bis(2-(4-((3,5-dibromobenzyl)oxy)phenylsulfonamido)-N-hydroxypentanamide) (**3**).*

Tetrahydropyranyl derivative **13** (70 mg, 0.054 mmol) was dissolved in DCM (6 mL) and TFA (0.5 mL, 6.15 mmol) was added dropwise at 0 °C. After 6 h of stirring, the solvents were evaporated and the residue was co-evaporated with DCM for three times. The crude was purified by flash chromatography using a Isolute Flash Si II cartridge ($CHCl_3$: MeOH 50:1) to give the desired hydroxamic acid **3** (31 mg, 55% yield). Mp 123-124 °C. ^{1}H NMR (400 MHz, DMSO-d_6) δ: 1.15-1.60 (m, 8H); 2.78-2.83 (m, 4H); 3.46-3.51 (m, 2H); 5.19 (s, 4H); 5.73 (t, *J*= 5.6 Hz, 2H); 7.14 (d, *J*= 8.8 Hz, 4H, Ar); 7.69-7.71 (m, 8H); 7.82 (m, 2H); 7.82-7.86 (m, 2H); 8.82 (br s, 2H); 10.52 (br s, 2H). ^{13}C NMR (100 MHz, DMSO-d_6) δ: 26.4; 28.3; 53.6; 67.8; 114.9; 122.6; 128.5; 129.5; 132.9, 133.8; 141.2; 158.0; 160.6; 167.3. Elemental analysis calcd (%) for $C_{37}H_{40}Br_4N_6O_{11}S_2$: C 39.38, H 3.57, N 7.45; found: C 39.42, H 3.57, N 7.47. HRMS (ESI) m/z calculated for $[C_{37}H_{40}Br_4S_2N_6O_{11}-H]^-$: 1122.8857, found: 1122.8875; calculated for $[C_{37}H_{40}Br_4S_2N_6O_{11}+Cl]^-$: 1158.8624, found 1158.8641.

IV. Crystallization and structure resolution

The human recombinant catalytic domain (F67D) MMP-12, available in our laboratory, was used for crystallogenesis experiment.[1] The MMP-12-**1** crystal complex was obtained by sitting drop vapour diffusion method, according with the crystallization procedure previously described.[2]

Briefly, 6 µL of MMP-12 solution at 380 µM concentration was added of 0.7 µL of compound **1** at 10 mM. The drops prepared with 1 µL of protein-inhibitor complex and 1 µL of 17% PEG 20000, 200 mM imidazole maleate, pH 8.5 and 250 mM NaCl. After the streak seeding, the crystals grew immediately and in few weeks they were suitable for data collection. The crystals were transferred to a pre-mixed cryo-protecting based on condition CM6 and 7 and CryoSol™ (Molecular Dimensions)[3] for a few seconds, then picked up into a loop and flash cooled in liquid nitrogen.

The data set for the MMP-12-**1** crystal complex was collected at the European Synchrotron Radiation Facility (ESRF) in Grenoble (France), on beamline ID23-1 from a single crystal and the data processed

automatically using XDS.[4] The crystal belongs to the space group *C*2 and diffracted up to 1.23 Å resolution with cell parameters a = 51.291 b = 60.39 c = 54.00; *β* = 115.23 (Table S1).

The structure was solved by molecular replacement MOLREP,[5] using as starting model the PDB structure code 4I03.[2] The structural model was visualized in COOT and refined in phenix.refine with several cycles.[6,7] The image was generated with PyMOL software.[8]

**Structure**	**MMP12-1**
**PDB code**	**7OVY**
Crystallization	17% PEG 20000, 0.2 M Imidazole Malate pH 8.5, 250mM NaCl
**Data Collection**	
Source	ESRF ID23-1
Wavelength (Å)	0.9763
Space group	*C*2
Unit-cell (Å)	a = 51.291, b = 60.39, c = 54.00; *β* = 115.23°
Molec./asym.	1
Resolution (Å)	50–1.23/1.24–1.23
$CC_{1/2}$ (%)	99.9/28.8
$\langle I/\sigma(I) \rangle$	10.66/0.80
No. of reflections	156501
No. of unique reflections	39823
Completeness (%)	92.6/64.6
Multiplicity	33.68/3.78
**Refinement**	**Phenix**
Resolution (Å)	48.98–1.23/1.24–1.23
No. of reflections	39913/39913 (non-anomalous)
R-work	18.27
R-free	21.3
RMSD Bond lengths (Å)	0.007
RMSD Bond angles (°)	1.208
Ramachandran favored	99.60%
Ramachandran outliers	0

Table S1. MMP-12-**1** crystal complex: crystallization, data collection, processing and refinement.

V. Molecular Modeling Studies

Docking Calculations. The crystal structure of ADAM8 (PDB code 4DD8) was taken from the Protein Data Bank.[9] After adding hydrogen atoms the structure was minimized using Amber 20 software and

parm03 force field at 300 K. The structure was placed in a rectangular parallelepiped water box, an explicit solvent model for water, TIP3P, was used and the complexes were solvated with a 20 Å water cap. Sodium ions were added as counter ions to neutralize the system. Two steps of minimization were then carried out; in the first stage, we kept the protein fixed with a position restraint of 500 kcal/mol•Å^2 and we solely minimized the positions of the water molecules. In the second stage, we minimized the entire system through 5000 steps of steepest descent followed by conjugate gradient (CG) until a convergence of 0.05 kcal/Å•mol. Automated docking was carried by means of the Gold program (version 5.1). The region of interest used by Gold was defined in order to contain the residues within 20 Å from the catalytic zinc ions. The “allow early termination” option was deactivated while the remaining Gold default parameters were used, and the ligand was submitted to 200 genetic algorithm runs by applying the ChemScore fitness function. The best docked conformation was taken into account. The General Amber Force Field (GAFF) parameters were assigned to the ligands. The partial charges were calculated using the AM1-BCC method, as implemented in the Antechamber suite of AMBER 20. The best docked conformation was taken into account.

MD Simulations. All simulations were performed using AMBER, version 20. MD simulations were carried out using the parm03 force field at 300 K. The complex was placed in a rectangular parallelepiped water box. An explicit solvent model for water, TIP3P, was used, and the complex was solvated with a 20 Å water cap. Sodium ions were added as counterions to neutralize the system. Prior to MD simulations, two steps of minimization were carried out using the same procedure described above. Particle mesh Ewald (PME) electrostatics and periodic boundary conditions were used in the simulation. The MD trajectories were run using the minimized structure as the starting conformation. The time step of the simulations was 2.0 fs with a cutoff of 10 Å for the nonbonded interaction, and SHAKE was employed to keep all bonds involving hydrogen atoms rigid. Constant-volume periodic boundary MD was carried out for 1.0 ns, during which the temperature was raised from 0 to 300 K. Then 550 ns for the ADAM8-JG26 complex and 1050 ns for the ADAM8-**1**-ADAM8 trimer of constant pressure periodic boundary MD were carried out at 300 K using the Langevin thermostat to maintain constant the temperature of our system. All the α carbons of the protein were blocked for the first 50 ns with a harmonic force constant of 10 kcal/mol•Å^2. General Amber force field (GAFF) parameters were assigned to the ligand, while partial charges were calculated using the AM1-BCC method as implemented in the Antechamber suite of AMBER 20. The final structure of the complexes

was obtained as the average of the last 500.0 ns of MD minimized by the CG method until a convergence of 0.05 kcal/mol•Å^2. The average structures were obtained using the ptraj program implemented in AMBER 20.

In silico physicochemical parameters.

Table S2. Predicted lipophilicity (logP), water solubility (logS) of compounds **1**-**3**.

Cpds	Cons. logP	Cons. logS
**1**	5.8	-13.7
**2**	5.7	-12.9
**3**	4.7	-11.3

VI. Biological Activity on Isolated Enzymes

MMP and ADAM10 inhibition assays.

Recombinant human MMP-14 catalytic domain was a kind gift of Prof. Gillian Murphy (Department of Oncology, University of Cambridge, UK). Pro-MMP-1, pro-MMP-2, and pro-MMP-9, were purchased from Calbiochem (Merck-Millipore). Pro-MMP-12 and ADAM10 were purchased from R&D Systems. Proenzymes were activated immediately prior to use with *p*-aminophenylmercuric acetate (APMA 2 mM for 1 h at 37°C for MMP-2 and MMP-1, 1 mM for 1 h at 37°C for MMP-9). Pro-MMP-12 was auto activated by incubating in the fluorometric assay buffer (FAB: Tris 50 mM, pH = 7.5, NaCl 150 mM, $CaCl_2$ 10 mM, Brij 35 0.05% and DMSO 1%) for 30 h at 37°C. For assay measurements, each inhibitor stock solution (DMSO, 10 mM) was further diluted in FAB at seven different concentrations. Activated enzyme (final concentration 0.56 nM for MMP-2, 1.3 nM for MMP-9, 1.0 nM for MMP-14cd, 2.0 nM for MMP-1, 2.3 nM for MMP-12 and 20 nM for ADAM10) and inhibitor solutions were incubated in the assay buffer for 3 h at 25 °C. ADAM10 was incubated for 1 h at 37°C in a different buffer at pH 9 (Tris 25 mM, $ZnCl_2$ 25 µM, Brij-35 0.005 %). After the addition of 200 µM solution of the fluorogenic substrate Mca-Lys-Pro-Leu-Gly-Leu-Dap(Dnp)-Ala-Arg-NH_2 (Bachem) for all the enzymes in DMSO (final concentration 2 µM for all enzymes, 10 µM for ADAM10), the hydrolysis was monitored every 10 sec. for 15 min. recording the increase in fluorescence (λ_{ex} = 325 nm, λ_{em} = 400 nm) using a Molecular Devices SpectraMax Gemini XPS plate reader. The assays were performed in triplicate in a total volume of 200 µL per well in 96-well microtitre plates (Corning, black, NBS). Control wells lack inhibitor. The MMP inhibition activity was expressed in relative fluorescent units

(RFU). Percent of inhibition was calculated from control reactions without the inhibitor. IC_{50} was determined using the formula: $v_i/v_o = 1/(1 + [I]/ IC_{50})$, where v_i is the initial velocity of substrate cleavage in the presence of the inhibitor at concentration [I] and v_o is the initial velocity in the absence of the inhibitor. Results were analyzed using SoftMax Pro software (version 5.4.3, Molecular Devices, Sunnyvale, CA) and Prism Software version 5.0 (GraphPad Software, Inc., La Jolla, CA, USA).

ADAM8 and ADAM17 inhibition assays

Recombinant ADAM8 and ADAM17 (kind gift of Franka Scharfenberg, University of Kiel) were diluted in assay buffer (25 mM Tris HCl, pH 8.0. 6x10-4 % Brij, 10 mM $CaCl_2$, 10 mM $ZnCl_2$) to a working concentration of 250 ng and incubated with inhibitors for 45 min in a total volume of 50 µL. The fluorescent substrate PepDAB13[10] was added in a concentration of 10 µM (in a volume of 50 µL). Fluorescence development was observed over 6 h at 37 °C in Fluostar Imager (BMG Labtech, Offenburg, Germany).

VII. Biological Activity on cells

Cell-based shedding assay

HEK293 cells stably transfected with ADAM8 and CD23 were incubated for 24 h at 37°C in the presence of inhibitors in concentrations indicated in DMEM medium supplemented with 10% fetal calf serum. A human soluble CD23 ELISA (R&D Systems, Biotechne, Wiesbaden) was used to detect ADAM8-dependent release of soluble CD23 in the cell culture medium. ELISA experiments were performed in three independent experiments in triplicates. The relative CD23 release (control = 100%) was determined for all samples.

Invasion assay

Thincerts of 8.0 µM pore size, translucent (Greiner BioOne) were transferred into 24-well plates. Each insert was loaded with 75 µL Matrigel (BD Biosciences), 1:1 diluted with serum-free medium DMEM. After 1 hour, thincerts were turned upside down and 25000 MDA-MB-231 cells, pretreated with vehicle (DMSO) or 20 µM inhibitors (JG26 and compounds **1-3** in DMSO) for 24 hours in DMEM/0.5 % FCS were seeded on the lower side and incubated for 3-4 hours so that cell can adhere to the membrane. To induce cell invasion, DMEM/20% FCS was applied to the upper chamber in order to

form a concentration gradient. After 18 hours, cells were fixed with 3.7 % paraformaldehyde and stained with 4',6-Diamidin-2-phenylindol (DAPI) to detect cell nuclei. Quantification of cell invasion was performed using a confocal microscope by stacked imaging. Cell nuclei present in the upper and the lower chamber were counted in 5 randomly chosen viewing fields and the amount of invasion was calculated in percent as the ratio between invaded cells/non-invaded cells x 100. Experiments were repeated 3 times, counting 5 random viewing fields per well, respectively.

Protein extraction and Western Blot analysis

After 48 h, total protein extraction was performed by detaching the cells with a cell scraper. Cells were washed with PBS, resuspended in 50 µL RIPA (50 mM HEPES pH 7.4; 150 mM NaCl; 1% (v/v) NP-40; 0.5% (w/v) Natriumdeoxycholate; 0.1% (w/v) SDS; 10 mM Phenantrolin; 10 mM EDTA; PierceTM Protease Inhibitor Mini Tablets, EDTA-free, Thermo Scientific; PierceTM Phosphatase Inhibitor Mini Tablets, Thermo Scientific) and incubated for 30 min on ice. After centrifugation at 12,000 x g for 5 min at 4 °C, the protein concentration of the supernatant containing the total protein was determined by PierceTM BCA Protein Assay Kit (Thermo Scientific). Proteins from equal amounts of lysate were then boiled in 1x loading buffer (5x loading buffer: 60 mM Tris-HCl pH 6.8; 2% (w/v) SDS; 10% (w/v) Glycerol; 5% (v/v) ß-Mercaptoethanol; 0.01% (w/v) Bromphenol-Blue) for 5 min. The proteins were separated by 10% SDS-PAGE and subsequently transferred on a nitrocellulose membrane (GE Healthcare). The membrane was then blocked with 5% (w/v) milk powder (MP) in TBST (50 mM Tris, pH 7.5; 150 mM NaCl; 0.1% (w/v) Tween-20) for 1 h and probed with the following primary antibodies: anti-beta Tubulin (NB600-936, Novus Biological, 1:2000), anti-MAPK (4696, Cell Signaling, 1:2000), anti-pMAPK (4370, Cell Signaling, 1:2000), anti-Akt (2920, Cell Signaling, 1:2000) and anti-pAkt (4060, Cell Signaling, 1:2000) at 4°C overnight. After washing the 3x with TBST, the membrane was incubated with horseradish peroxidase-conjugated secondary antibodies for 1 h (abcam, 1:5000) followed by a repeated washing step. Detection was performed by using Chemiluminescent HRP Substrate, Western Bright Sirius (Advansta).

VIII. Representative NMR spectra

Compound **1**: ^{1}H NMR (400 MHz, CD_3OD)

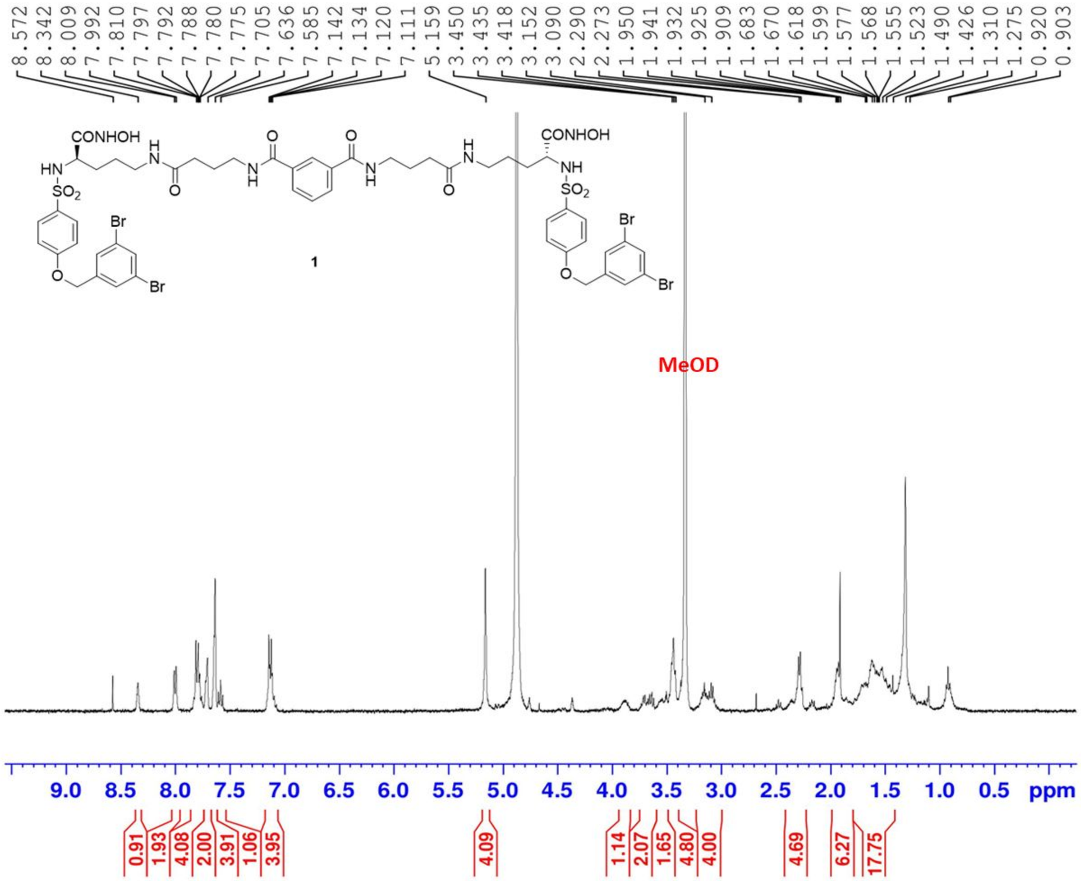

Compound **1:** ^{13}C NMR (CD_3OD)

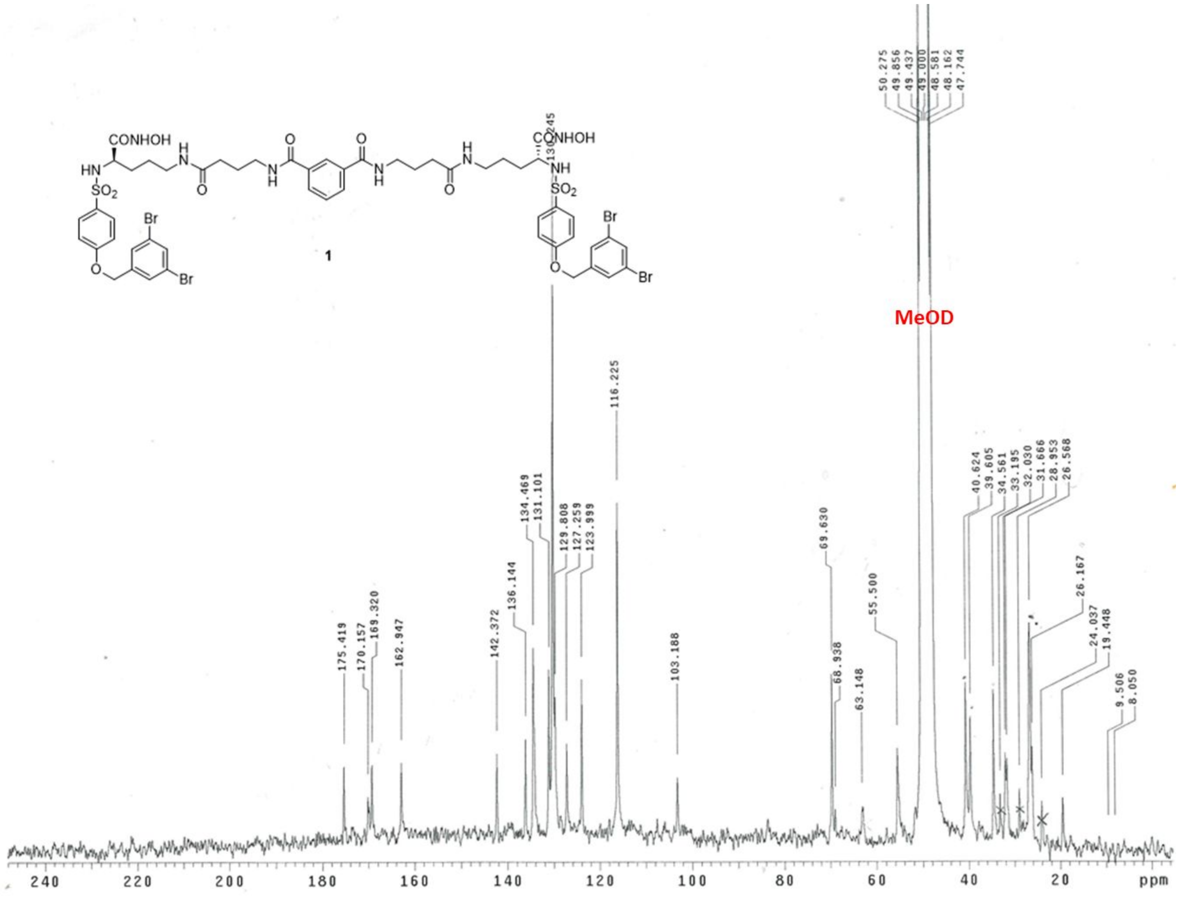

Compound **2**: ^{1}H NMR (400 MHz, CD_3OD)

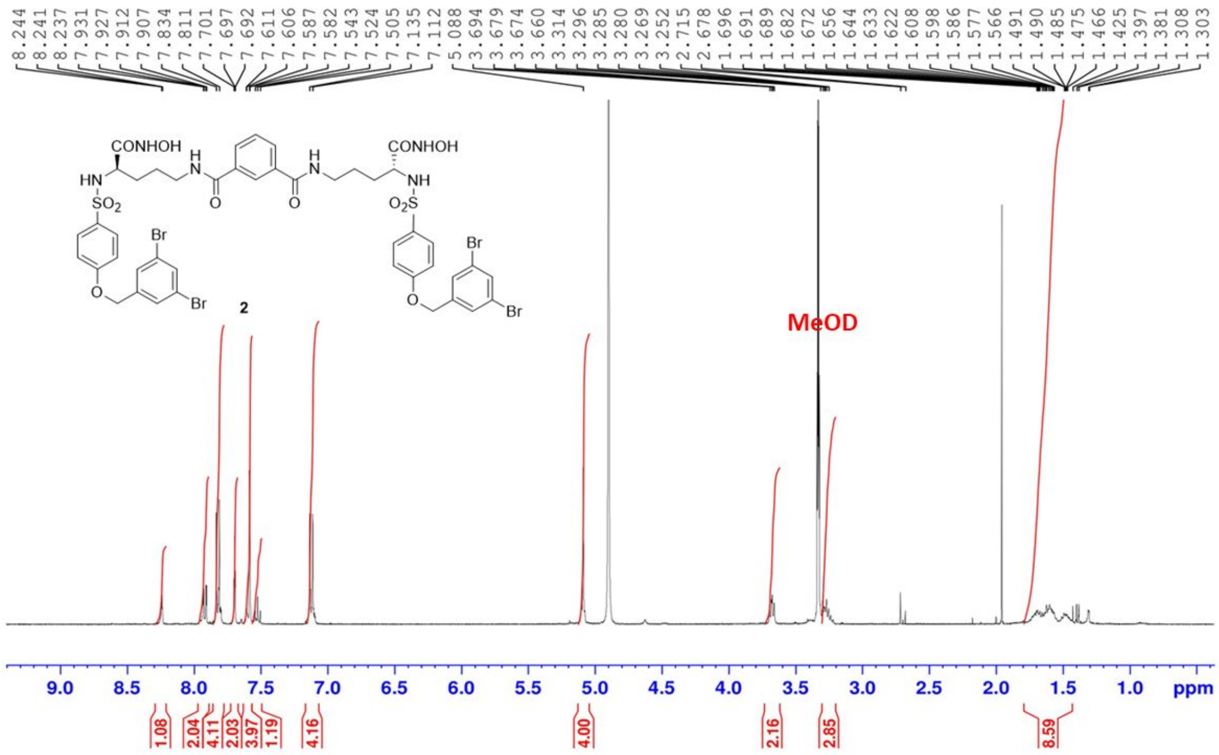

Compound **2**: ^{13}C NMR (100 MHz, CD_3OD)

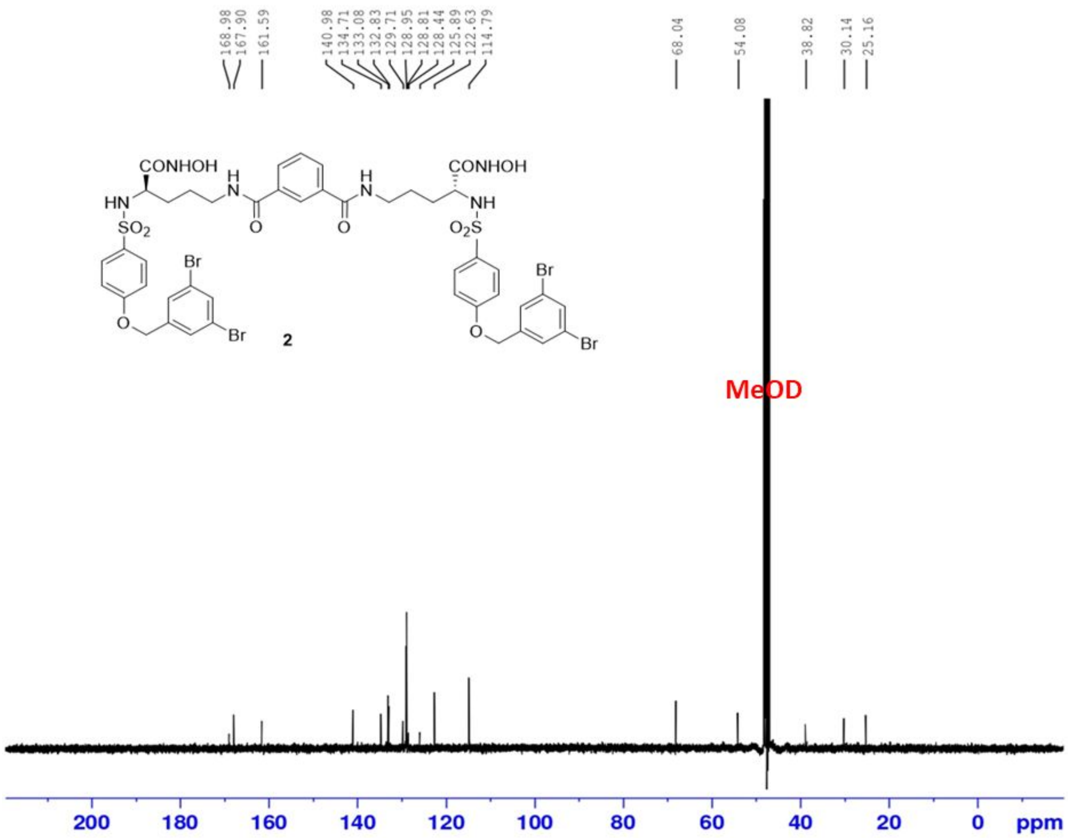

Compound **3**: ^{1}H NMR (400 MHz, DMSO-d_6).

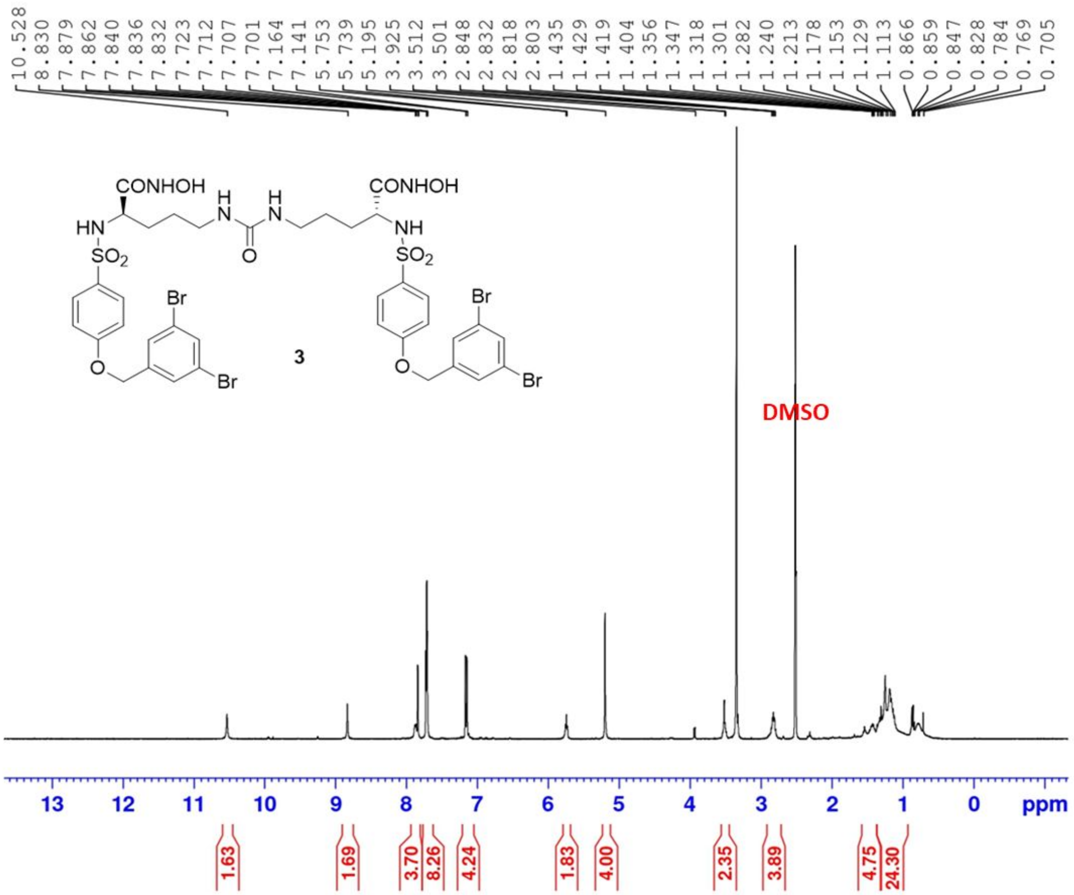

Compound **3**: ^{13}C NMR (100 MHz, DMSO-d_6).

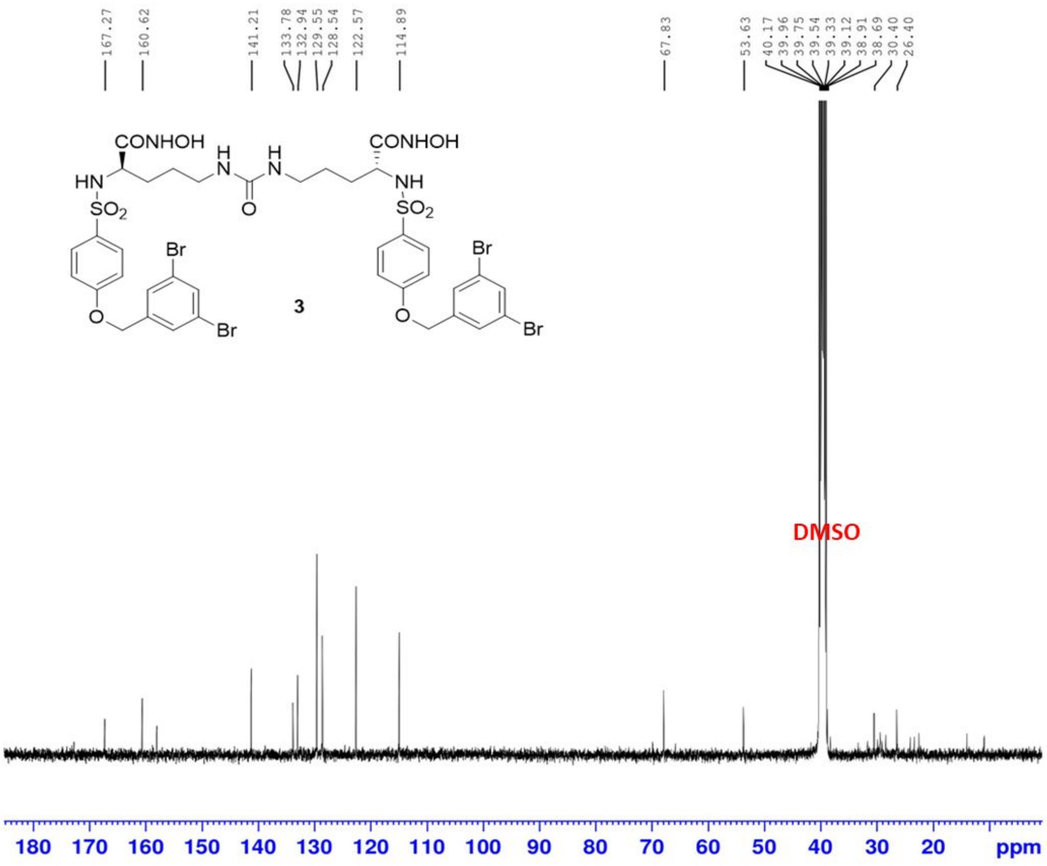

IX. Supporting Information References

---

[1] Vera, L., Stura, E. A. Strategies for protein cryocrystallography. *Cryst. Growth Des.* **2014** *14* (2), 427-435. http://dx.doi.org/10.1021/cg301531f.

[2] Antoni C, Vera L, Devel L, Catalani MP, Czarny B, Cassar-Lajeunesse E, Nuti E, Rossello A, Dive V, Stura EA. Crystallization of bi-functional ligand protein complexes. *J Struct Biol*. **2013**, 182(3):246-54. doi: 10.1016/j.jsb.2013.03.015.

[3] Ciccone L, Vera L, Tepshi L, Rosalia L, Rossello A, Stura EA. Multicomponent mixtures for cryoprotection and ligand solubilization. *Biotechnol Rep* (Amst). **2015**; 7:120-127. doi: 10.1016/j.btre.2015.05.008.

[4] Kabsch W. Integration, scaling, space-group assignment and post-refinement. *Acta Crystallogr D Biol Crystallogr.* **2010**, 66(Pt 2):133-44. doi: 10.1107/S0907444909047374.

[5] Vagin A, Teplyakov A. Molecular replacement with MOLREP. *Acta Crystallogr D Biol Crystallogr.* **2010**;66(Pt 1):22-5. doi: 10.1107/S0907444909042589.

[6] Afonine PV, Grosse-Kunstleve RW, Echols N, Headd JJ, Moriarty NW, Mustyakimov M, Terwilliger TC, Urzhumtsev A, Zwart PH, Adams PD. Towards automated crystallographic structure refinement with phenix.refine. *Acta Crystallogr D Biol Crystallogr.* **2012**; 68(Pt 4):352-67. doi: 10.1107/S0907444912001308.

[7] Emsley P, Lohkamp B, Scott WG, Cowtan K. Features and development of Coot. *Acta Crystallogr D Biol Crystallogr.* **2010**;66(Pt 4):486-501. doi: 10.1107/S0907444910007493.

[8] PyMOL Molecular Graphics System, version 2.3.2; Schrödinger LLC, 2020.

[9] Berman, H. M.; Westbrook, J.; Feng, Z.; Gilliland, G.; Bhat, T. N.; Weissig, H.; Shindyalov, I. N.; Bourne, P. E. The Protein Data Bank. *Nucleic Acids Res.* **2000**, *28*, 235-242.

[10] Schlomann U, Dorzweiler K, Nuti E, Tuccinardi T, Rossello A, Bartsch JW. Metalloprotease inhibitor profiles of human ADAM8 in vitro and in cell-based assays. *Biol Chem*. **2019**, 400, 801-810.
